# Genome-wide association study on serum alkaline phosphatase levels in a Chinese population

**DOI:** 10.1186/1471-2164-14-684

**Published:** 2013-10-05

**Authors:** Jun Li, Lixuan Gui, Chen Wu, Yunfeng He, Li Zhou, Huan Guo, Jing Yuan, Binyao Yang, Xiayun Dai, Qifei Deng, Suli Huang, Lei Guan, Die Hu, Siyun Deng, Tian Wang, Jiang Zhu, Xinwen Min, Mingjian Lang, Dongfeng Li, Handong Yang, Frank B Hu, Dongxin Lin, Tangchun Wu, Meian He

**Affiliations:** 1 MOE Key Lab of Environment and Health, School of Public Health, Tongji Medical College, Huazhong University of Science & Technology, 430030 Wuhan, Hubei, China; 2 State Key Laboratory of Molecular Oncology, Cancer Institute & Hospital, Chinese Academy of Medical Sciences and Peking Union Medical College, 100021 Beijing, China; 3 Dongfeng Central Hospital, Dongfeng Motor Corporation and Hubei University of Medicine, 442008 Shiyan, Hubei, China; 4 Departments of Nutrition and Epidemiology, Harvard School of Public Health, Boston, MA 02115, USA; 5 Department of Epidemiology, School of Public Health and Management, Chongqing Medical University, 400016 Chongqing, China

**Keywords:** Genetic variations, Serum alkaline phosphatase, Heterogeneity, GWAS, Gene-environment interaction

## Abstract

**Background:**

Serum alkaline phosphatase (ALP) is a complex phenotype influenced by both genetic and environmental factors. Recent Genome-Wide Association Studies (GWAS) have identified several loci affecting ALP levels; however, such studies in Chinese populations are limited. We performed a GWAS analyzing the association between 658,288 autosomal SNPs and serum ALP in 1,461 subjects, and replicated the top SNPs in an additional 8,830 healthy Chinese Han individuals. The interactions between significant locus and environmental factors on serum ALP levels were further investigated.

**Results:**

The association between *ABO* locus and serum ALP levels was replicated (*P* = 2.50 × 10^-21^, 1.12 × 10^-56^ and 2.82 × 10^-27^ for SNP rs8176720, rs651007 and rs7025162 on *ABO* locus, respectively). SNP rs651007 accounted for 2.15% of the total variance of serum ALP levels independently of the other 2 SNPs. When comparing our findings with previously published studies, ethnic differences were observed across populations. A significant interaction between *ABO* rs651007 and overweight and obesity was observed (*FDR* for interaction was 0.036); for individuals with GG genotype, those with normal weight and those who were overweight or obese have similar serum ALP concentrations; minor allele A of rs651007 remarkably reduced serum ALP levels, but this effect was attenuated in overweight and obese individuals.

**Conclusions:**

Our findings indicate that *ABO* locus is a major determinant for serum ALP levels in Chinese Han population. Overweight and obesity modifies the effect of *ABO* locus on serum ALP concentrations.

## Background

Alkaline phosphatases (ALP), which is essential in bone mineralization and functions in vascular calcification [1], is a group of hydrolytic isoenzymes with low substrate specificity that catalyze the hydrolysis of organic phosphate esters at an alkaline environment [2]. Four genes encode ALP, including tissue-nonspecific ALP gene located on 1p36.12, which is expressed in various tissues such as osteoblasts, hepatocytes, kidney and early placenta; and three tissue-specific ALP genes located on 2q37, which are expressed in placenta, germ cells, and intestine, respectively [3]. In healthy individuals, serum ALP derives mostly from liver/bone/kidney, partially from intestine especially during postprandial time or in blood type O or B secretors, and little is contributed from placenta in the third trimester of pregnancy [4,5]. The concentration of serum ALP is elevated in pathological conditions like osteoblast activated bone disorders, bile-flow obstructions, tumor metastases, liver diseases, leukemia, hyperthyroidism, infections and obesity, while lower level occurs in anemia and hypothyroidism [2,6]. Current evidence demonstrated that elevated serum ALP levels were associated with adverse outcomes of dialysis patients and increased all-cause death rate in myocardial infraction survivors [7-9]. In particular, positive associations between ALP levels and all-cause death rate together with cardiovascular mortality were also observed in the general population [9]. Therefore, investigating the underlying factors that influence serum ALP levels are meaningful for both clinical medicine and public health.

Serum ALP is a complex trait influenced by both polygenic and environmental factors. It is reported that age, gender, smoking, diet, body mass index (BMI), and physical activities could affect serum ALP concentrations [4,10-13]. The additive genetic heritability of serum ALP in mice is approximately 56% [14]. Recent GWAS have identified several loci, including *NBPF3-ALPL*, *ABCB11*, *GPLD1*, *PPP1R3B*, *TRIB1*, *ABO*, *JMJD1C*, *REEP3*, *FADS2*, *PMFBP1*, *DLG4*, *FUT2* and *ABHD12*, associated with serum ALP levels in European ancestry populations, Asian-Indians and Japanese populations [15-17], however, such researches in populations of Chinese origin are limited, and no study investigated gene-environment interactions on serum ALP levels. In the present study, we performed a GWAS in 1,461 subjects and replicated the top SNPs in additional 8,830 healthy Chinese Han individuals. The purposes of the study were to (i) detect genetic determinants for serum ALP in Chinese Han population; (ii) compare the present results with published findings to seek heterogeneity among Chinese and other populations and (iii) investigate the gene-environment interactions on serum ALP levels.

## Results

### Characteristics of the study population

A total of 1,452 subjects (21.8% female, 63.05 ± 8.14 years old) were included in the discovery set with a mean (SD) serum ALP concentration of 90.02 (26.51) U/L; validation were conducted in 8,830 individuals (58.2% female, 61.95 ± 7.83 years old) whose mean (SD) serum ALP concentration was 90.26 (35.41) U/L. The demographics of the study population together with information on potential influencing factors of serum ALP are summarized in Additional file 1: Table S1.

### Association with alkaline phosphatase level

**Figure 1:**
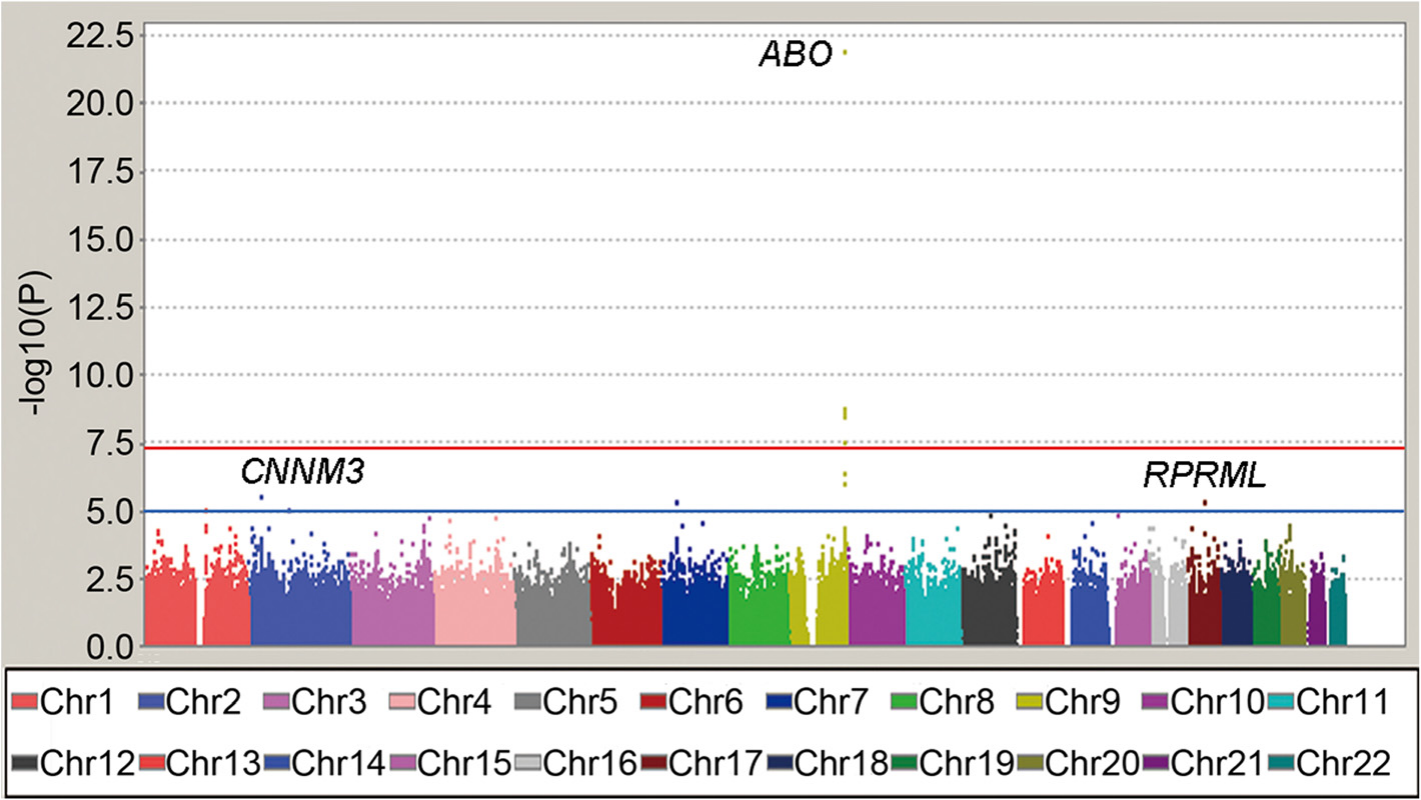
**Manhattan plot of genome-wide association analyses for ALP.** The horizontal axis shows the chromosomal positions while the vertical axis shows -log10 *P* values from the test of association by linear regression analysis. The red horizontal line shows the *P* value of 5.0 × 10^-8^, and the blue horizontal line corresponds to a *P* value of 1.0 × 10^-5^.

**Figure 2:**
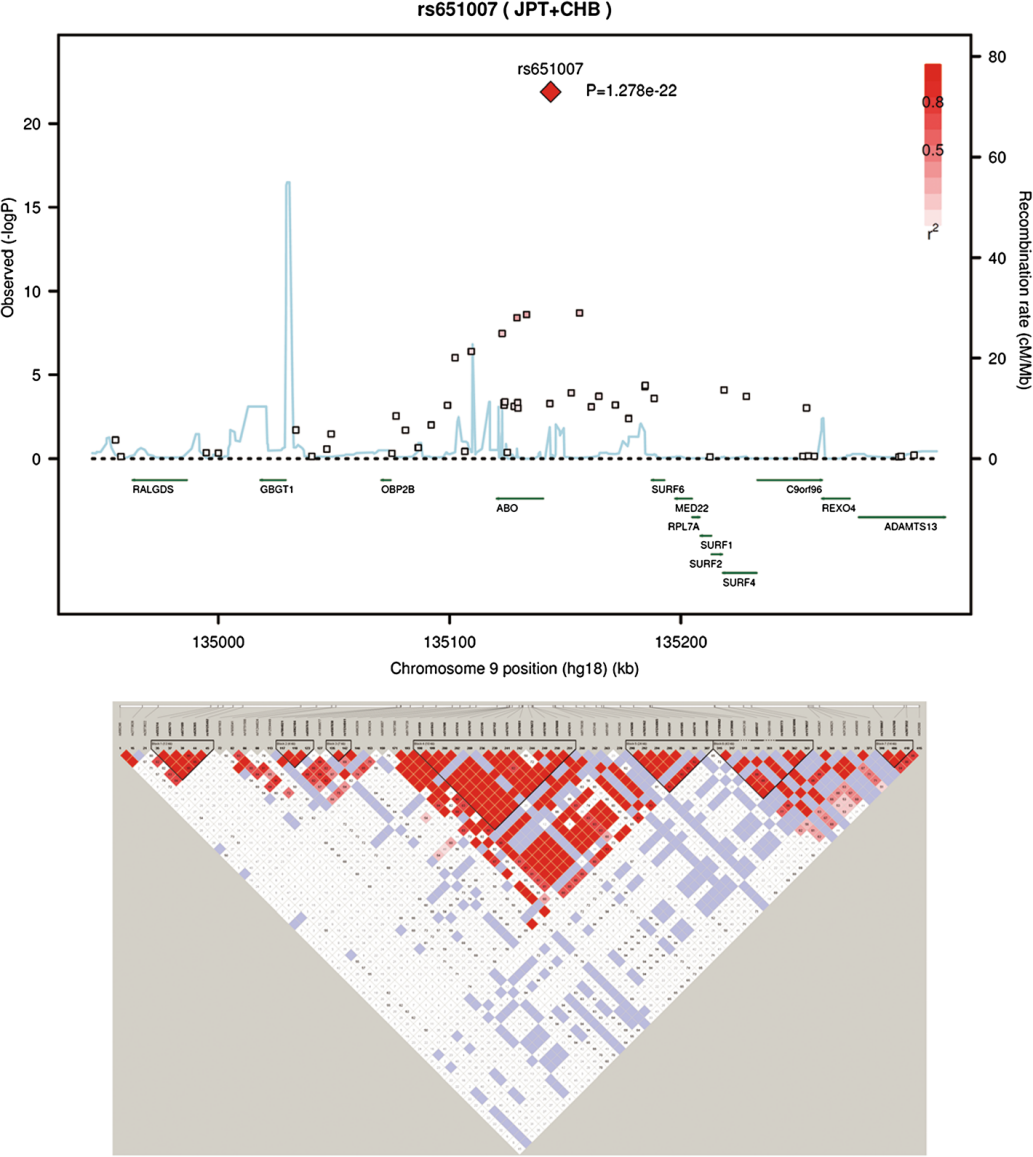
**Regional association plot on 9q34 for SNP-ALP associations.** The horizontal axis shows the chromosomal positions in the NCBI build 36 genome sequences. In the upper part of the figure, the black dots represent *P* values of SNPs genotyped by Affymetrix SNP array. The sky blue line shows the recombination rates given by the Hapmap release 22. The middle green arrows indicate the RefSeq genes. The lower part of the figure shows a LD map of r^2^ values drawn by the Haploview software (http://www.broadinstitute.org/haploview/haploview) using release 22 HapMap genotype data of the Chinese (CHB) and Japanese (JPT).

In GWAS stage, a total of 906,703 SNPs were genotyped among 1,461 subjects in which 38,446 SNPs not mapped on autosomes were excluded. After QC filtering, SNPs with minor allele frequency (MAF) < 0.01, Hardy-Weinberg Equilibrium (HWE) < 0.0001, and SNPs call rate < 95% were excluded. Individuals with a call rate < 95% were also ruled out for further analysis. Finally, 1,452 subjects with 658,288 autosomal SNPs were retained for association analyses, with an overall call rate of 99.68%. Quantile - Quantile plot revealed a good match between the distributions of the observed *P* values and those expected by chance, and a genomic inflation factor of 1.011 suggested that population stratification effects were negligible in our study samples (Additional file 2: Figure S1). The Manhattan plot showed that 2p11.2 (lead SNP rs7594727, *P* = 3.70 × 10^-5^), 2p23.1 (lead SNP rs1383023, *P* = 2.30 × 10^-6^), 9q34.2 (lead SNP rs651007, *P* = 1.93 × 10^-21^) and 17q21.32 (lead SNP rs7214920, *P* = 2.55 × 10^-6^) were associated with serum ALP at 5.0 × 10^-5^ significance level (Figure 1, Table 1). The regional association plot revealed the SNP-ALP associations and linkage disequilibrium (LD) relations on 9q34.2 (Figure 2). The present study has 91.3% power to detect SNPs that account for 2% of the ALP variance at 5 × 10^-5^ significance level; however, it has only 48.6% power to detect SNPs at 5 × 10^-8^ significance level, hence 5 × 10^-5^ was set to be the cutoff point in SNP selection. Seven SNPs met the selection criteria (see Method). Among them, rs657152 on 9q34.2, which has been reported to be associated with ALP in European and Japanese population [16,17], was replicated with a *P* value of 5.88 × 10^-9^ in our GWAS set. Therefore, we did not validate this SNP in the second stage. The other 6 SNPs were selected for further validation.

**Table 1:** SNPs associated with serum ALP levels

**SNP**	**Region**	**Position**^**a**^	**Genes**	**Locus**	**Minor/major allele**	**GWAS**^**b**^			**Validation**^b*^			**Combined**^**b**^		
						**MAF**	**Effect size (s.e.m)**	***P *values**	**MAF**	**Effect size (s.e.m)**	***P *values**	**MAF**	**Effect size (s.e.m)**	***P *values**
rs1383023	2p23.1	31872045	*N/A*	**-**	A/C	0.093	0.087 (0.018)	2.30 × 10^-6^	0.075	0.0016 (0.014)	9.05 × 10^-1^	0.080	-0.0009(0.005)	8.59 × 10^-1^
rs7594727	2p11.2	96853597	*CNNM3*	Intron	A/C	0.107	-0.070 (0.017)	3.70 × 10^-5^	0.116	-0.008 (0.011)	5.01 × 10^-1^	0.113	-0.018(0.009)	4.39 × 10^-2^
rs8176720	9q34.2	135122694	*ABO*	Exon	C/T	0.444	0.055 (0.010)	8.19 × 10^-8^	0.454	0.038 (0.005)	6.63 × 10^-16^	0.452	0.041(0.004)	2.50 × 10^-21^
rs651007	9q34.2	135143696	*SURF6-ABO*	Up-	A/G	0.244	-0.114 (0.012)	1.93 × 10^-21^	0.222	-0.077 (0.005)	7.54 × 10^-44^	0.226	-0.079(0.005)	1.12 × 10^-56^
rs7025162	9q34.2	135156167	*SURF6-ABO*	Up-	A/G	0.390	-0.064 (0.011)	2.20 × 10^-9^	0.383	-0.044 (0.005)	1.10 × 10^-20^	0.384	-0.047(0.004)	2.82 × 10^-27^
rs7214920	17q21.32	42406128	*RPRML*	Up-	A/G	0.119	0.068 (0.016)	2.55 × 10^-6^	0.119	0.001 (0.011)	9.12 × 10^-1^	0.119	0.020(0.009)	2.99 × 10^-2^

^a^Genome position is based on NCBI build 36.3.

^b^The *P* values in GWAS stage are based on linear regression analysis on the log-transformed ALP with adjustment for age, gender and the top two eigenvectors in PCA analysis assuming an additive model by PLINK. The validation and combined *P* values are calculated by linear regression model on the log-transformed ALP and adjusted for age and gender.

^*^SNPs of rs8176720, rs651007 and rs7025162 were validated in 8,830 healthy participants, while rs1383023, rs7594727 and rs7214920 were validated in 3,456 participants.

Three SNPs located within or near the *ABO* locus showed significant association with serum ALP in validation population (*P* = 6.63 × 10^-16^, *P* = 7.54 × 10^-44^ and *P* = 1.10 × 10^-20^ for rs8176720, rs651007, and rs7025162, respectively) (Table 1). Using each SNP as a covariate, we performed conditional analysis on the remaining 2 SNPs for their association with serum ALP. The results revealed that rs651007 was associated with serum ALP levels (*P* = 1.84 × 10^-23^) independently of the other two SNPs, and it accounted for 2.15% of the total variance of serum ALP (Additional file 3: Table S2). A significant difference in ALP levels among different genetic-inferred ABO blood groups was observed in GWAS dataset (ALP = 79.00 U/L, 80.86 U/L, 91.16 U/L and 92.82 U/L in individuals with blood group A, AB, B and O, *P* = 1.57 × 10^-18^), similar to previous reports [18,19]. To further explore whether the effect of the lead SNP rs651007 was driven by ABO blood group, we introduced ABO blood group into the multivariable linear regression model. As expected, the association between ALP levels and rs651007 dramatically reduced after ABO blood group adjustment. (β = - 0.036, *P* = 0.029, data not shown). Other three loci that mapped on 2p23.1 (rs1383023, *P* = 0.905), 2p12.2 (rs7594727 in *CNNM3, P* = 0.501) and 17q21.32 (rs7214920 close to *RPRML, P* = 0.912) failed to be replicated (Table 1). The unbalanced distribution of the demographic characteristics in the discovery and replication samples in the present study might partly contribute to the lack of replication.

### Genetic heterogeneity on ALP levels among studies

GWAS results of the present study were compared with previously published studies that conducted in populations of European and Japanese origins [15-17] (Table 2). Although *ABO* locus found in other populations has been replicated in the present study, other loci identified in one or two earlier studies failed to be replicated. We did not observe positive signals on *ALPL* or *GPLD1*, which has been reported in European and Japanese studies (for *ALPL*, *P* for lead SNP is 7.0 × 10^-15^, 5.08 × 10^-13^ and in 3.70 × 10^-2^ in European study, Japanese study and the present study, respectively; for *GPLD1*, *P* for lead SNP is 1.2 × 10^-11^, 2.13 × 10^-11^ and in 1.61 × 10^-3^ in European study, Japanese study and the present study, respectively). Many loci identified in populations of European origin, e.g. *ABCB11*, *PPP1R3B*, *C9orf125*, *REEP3*, *ST3GAL4* and *ABHD12*, are neither significant in Japanese populations nor in our population although some of them got comparable effect sizes across studies (e.g. for rs12355784 on *JMJD1C*, *P* = 5.0 × 10^-10^, 8.66 × 10^-3^ and 1.60 × 10^-2^, and effect size = 0.025, 0.049 and 0.029 in European study, Japanese study and the present study, respectively). Only rs7173947, which mapped on an intergenic region on chromosome 15, has been identified in Japanese population with no significant signals around the locus being seen in European or in the present study. The heterogeneity test showed considerable heterogeneity across studies for some SNPs (e.g. for rs2242420 on *ALPL*, *P* of Q = 2.61 × 10^-6^, I^2^ = 0.955), which may attribute to ethnic heterogeneity and differences in characteristics of samples and study designs, etc. The small sample size also limited the power of the present study to replicate SNPs with low MAF and/or minor effects (for SNPs that explain 1.0% of the ALP variance with MAF of 0.05, a sample size of 2,400 is needed to detect the association with 80% discovery power at 5 × 10^-5^ significance level).

**Table 2:** Ethnic differences in major genetic variants associated with serum ALP levels

**SNP**	**Gene**	**Chr**	**Effect allele**	**Present study (Chinese)**^**a**^			**Japanese**^**b**^			**CEU**^**b**^			***P *value of Q**	**I**^**2**^	**No. of studies**
				**EAF**	**Effect size**	***P *value**	**EAF**	**Effect size**	***P *value**	**EAF**	**Effect size**	***P *value**			
rs1780324	*NBPF3-ALPL*	1	T	0.29	0.020	9.56 × 10^-2^	0.28	0.075	7.14 × 10^-5^	0.44	0.031	7.0 × 10^-15^	0.051	0.664	3
rs2242420	*ALPL*	1	T	0.20	-0.032	3.70 × 10^-2^	0.18	0.163	5.08 × 10^-13^	0.14	-	-	2.61 × 10^-6^	0.955	2
rs16856332	*ABCB11*	2	G	0.06	-0.026	0.270	0.07	-	-	0.39	0.031	1.6 × 10^-9^	0.638	0.000	2
rs9467160	*GPLD1*	6	A	0.03	-0.032	0.369	0.003	-0.05	0.76	0.21	0.034	1.2 × 10^-11^	0.171	0.433	3
rs6911965	*GPLD1*	6	C	0.11	-0.056	1.61 × 10^-3^	0.66	-0.235	2.13 × 10^-11^	0.17	-	-	5.35 × 10^-6^	0.952	2
rs6984305	*PPP1R3B*	8	A	0.01	0.010	0.863	0	-	-	0.11	0.027	2.1 × 10^-10^	0.772	0.000	2
rs2954021	*TRIB1*	8	A	0.41	0.035	2.51 × 10^-3^	0.43	-	-	0.50	0.014	2.3 × 10^-13^	0.095	0.641	2
rs10819937	*C9orf125*	9	C	0.36	0.020	0.114	0.32	-	-	0.17	0.025	1.0 × 10^-9^	0.741	0.000	2
rs657152	*ABO*	9	T	0.46	-0.062	3.78 × 10^-8^	0.44	-0.223	1.35 × 10^-38^	0.38	-0.047	1.7 × 10^-30^	0	0.975	3
rs12355784	*JMJD1C*	10	C	0.33	0.029	1.60 × 10^-2^	0.50	0.046	8.66 × 10^-3^	0.49	0.025	5.0 × 10^-10^	0.558	0.000	3
rs10761779	*REEP3*	10	G	0.37	0.031	7.83 × 10^-3^	0.45	-	-	0.49	0.025	6.9 × 10^-10^	0.634	0.000	2
rs174601	*FADS2*	11	A	0.41	0.007	0.563	0.38	-	-	0.35	0.017	2.6 × 10^-9^	0.075	0.684	2
rs2236653	*ST3GAL4*	11	T	0.70	-0.024	4.40 × 10^-2^	0.60	-	-	0.42	0.015	1.8 × 10^-9^	0.488	0.000	2
rs7173947	*intergenic*	15	C	0.33	-0.010	0.391	0.29	-0.105	3.23 × 10^-8^	0.41	-	-	2.75 × 10^-5^	0.943	2
rs7186908	*PMFBP1*	16	C	0.22	-0.028	4.73 × 10^-2^	0.34	-	-	0.24	0.02	4.8 × 10^-9^	1.35 × 10^-3^	0.903	2
rs314253	*DLG4*	17	G	0.51	0.005	0.658	0.52	-	-	0.33	0.021	8.4 × 10^-12^	0.179	0.447	2
rs281377	*FUT2*	19	T	0.86	0.045	2.56 × 10^-2^	0.87	-	-	0.43	0.018	1.1 × 10^-15^	2.42 × 10^-3^	0.891	2
rs7267979	*ABHD12*	20	G	0.07	-0.031	0.173	0.10	-	-	0.57	0.015	7.4 × 10^-10^	0.484	0.000	2

^**a**^In order to get genotypes of all reported SNPs, imputed genotype data were used in the presented ethnic comparison table. EAF is short for effect allele frequency.

^**b**^Quoted from Chambers, et al. [15], Kamatani, et al. [16] and Yuan, et al. [17].

### Gene-environment interaction analysis

**Figure 3:**
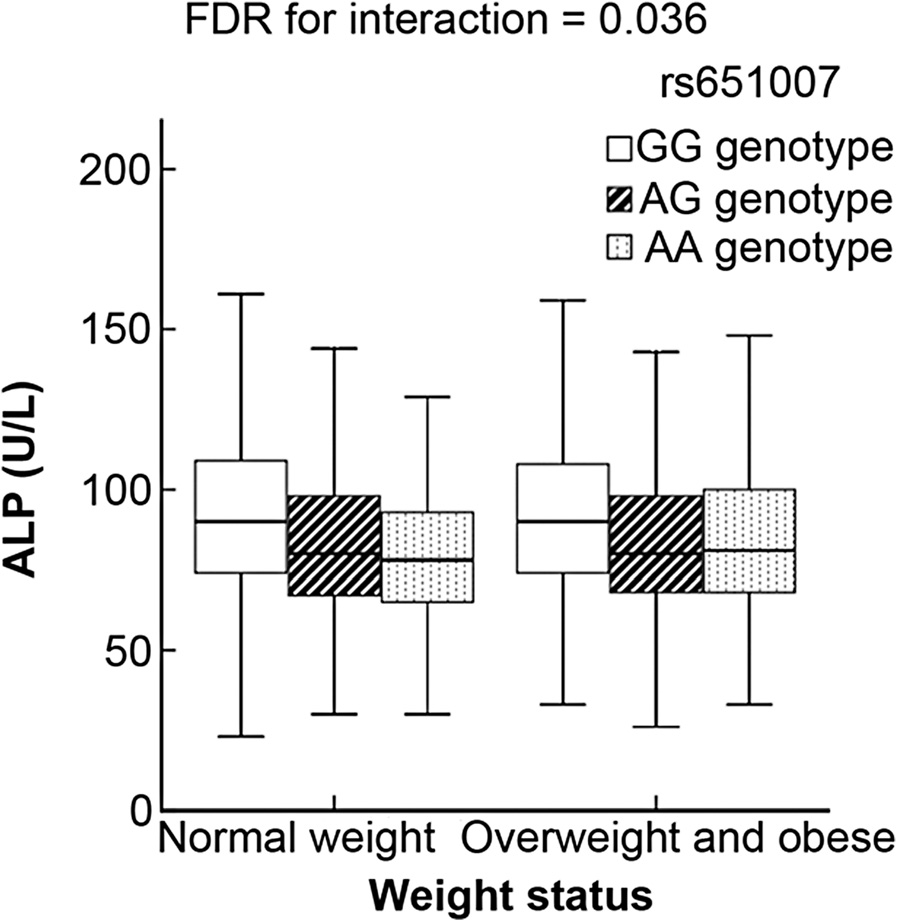
**Box plot for interaction between rs651007 genotypes and overweight and obesity.** The vertical axis shows serum ALP concentrations while the horizontal axis shows the BMI status (normal weight/overweight and obese). Minor allele A diminished serum ALP levels but this effect was attenuated in overweight or obese individuals.

Multiple factors have been proved to influence serum ALP levels, including age, gender, BMI, smoking, drinking and physical activity [10-13,20]. Therefore, we tested whether the independent SNP rs651007 interacted with age, gender, BMI (normal/overweight or obese), smoking or drinking on affecting ALP concentrations. As shown in Additional file 4: Table S3, rs651007 had a significant interaction with overweight and obesity on serum ALP levels (*FDR* = 0.036). For GG genotype carriers, the mean of serum ALP were similar between those with normal weight and those who were overweight or obese (89.52 U/L in overweight and obese subjects vs. 89.93 U/L in subjects with normal weight); GA or AA genotype carriers have significantly decreased serum ALP concentrations compared with GG genotype carriers; however, this effect was attenuated in overweight or obese individuals (β for rs651007 = -0.091 and -0.069 in subjects with normal weight and subjects who were overweight or obese, respectively). Thus, among AA genotype carriers, overweight or obese individuals had higher ALP levels than those with normal weight (81.24 U/L in overweight and obese subjects vs. 77.93 U/L in normal weight subjects) (Figure 3). The statistic power of interaction analysis in the present study is 71.1%. No significant gene-environment interactions were observed between rs651007 and other factors including age, gender, drinking, and smoking (Additional file 4: Table S3).

## Discussion

In the present two-stage GWAS, we confirmed that previously identified locus *ABO* was associated with serum ALP levels in a Chinese population. When comparing the present study with previously published findings, ethnic differences were observed across populations. In addition, a significant interaction between overweight and obesity and *ABO* rs651007 on serum ALP was found. The effects of rs651007 on serum ALP levels were attenuated in overweight or obese individuals than in normal weight subjects.

The association between *ABO* locus and serum ALP has been reported in other populations [15-17]. The present study was consistent with previous findings that serum ALP levels are much higher in individuals with blood type B or O [18]; the lead SNP rs651007 on *ABO* locus, which is in high LD with previously reported rs495828 (r^2^ = 0.916), accounted for 2.15% of the total variance of serum ALP in our population, similar with that reported in earlier studies (2.0% for rs657152 in Europeans and 3.79% for rs495828 in Japanese) [16,17]. The coding product of human *ABO* gene is a glycosyltransferase, of which catalytic activity could facilitate the transfer of carbohytrates to H antigen to form the antigenic structure of the ABO blood groups [21]. Current evidence found that human ABO blood groups or genetic variations on *ABO* gene were associated with the risk of various diseases, such as pancreatic cancer, gastric cancer, falciparum malaria, venous thromboembolism, myocardial infarction and type 2 diabetes [22-28]. Genetic variations on *ABO* gene were also found to be related with levels of serum E-selectin, sICAM, RBC, Hb, vWF, and CEA [16,25,29]. In healthy fasted individuals, approximately 90% of serum ALP originate from liver/bone/kidney, nearly 10% from intestine and in some cases 1% from placenta [5]. Most of the intestinal ALP is attached to ABO antigens on the surface of erythrocytes by a glycosyl-phosphatidylinositol anchor. Erythrocytes of blood type A bind to almost all intestinal ALP, while erythrocytes of blood type B/O bind to a much lesser degree, therefore results in a more prevalent presence of intestinal ALP in serum in individuals with blood type B/O [19,30]. In this sense, the differences in the binding capacity between intestinal ALP and erythrocytes among individuals with different blood groups might be a potential mechanism underlying the association between ALP and ABO locus identified in GWAS. The observation that association between rs651007 and serum ALP dramatically reduced after ABO blood group adjustment also lend support to this deduction, indicating that ABO blood groups may act as the driving force behind this association.

Differences were observed among studies when comparing our findings with previously published results and parts of the reason might be due to the ethnic heterogeneity, such as varied effect allele frequencies and unique LD structures. For example, the MAF of rs16856332 are 0.39, 0.06 and 0.07 in Europeans, Chinese and Japanese, respectively; the MAF of rs9467160 on *GPLD1* locus is 0.21 in European but is only 0.03 in Chinese Han, and even lower, 0.003 in Japanese; the scenario is the same for rs7267979 on *ABHD12* that its effect allele frequency is 0.57 in European but is only 0.07 in Chinese. On the contrary, rs281377 has a MAF of 0.43 in European but the frequency of this effective allele is 0.86 in Chinese. Meanwhile, not all *ABO* SNPs identified in Europeans are significant in Chinese, which may partially due to the differences in LD structure, e.g. the LD relation between rs8176720 and rs514708 on *ABO* gene is weak in Asian populations, but get stronger in Europeans (r^2^ = 0.38, 0.36 and 0.67 in the present, Japanese and European populations). Unique population structures, including different modifier genes, gene-gene or gene-environment interactions, different lifestyles or environment exposures can also be explanations for the heterogeneity across populations [31]. Notably, although it showed no evidence for heterogeneity across studies for SNPs on *ABCB11*, *PPP1R3B*, *C9orf125*, *JMJD1C*, *REEP3*, *ST3GAL4*, and *ABHD12*, SNPs on *ALPL*, *GPLD1*, *PMFBP1* and *FUT2* represented considerable heterogeneity among studies. The lack of replication of many loci may also due to different sample selection criteria, different study designs and statistical analysis methods, as well as the relatively small sample size of the GWAS stage of the present study. Studies with larger sample size, different ethnic sources and multi-center cooperations are needed to explain the ethnic differences better.

Previous study found that serum ALP was higher in obese patients [32]. The interaction between rs651007 and overweight and obesity on ALP levels found in the present study may be one of the explanations. The effect of rs651007 on serum ALP was attenuated in overweight or obese individuals compared with normal weight subjects, resulting in higher ALP levels in overweight or obese individuals. eQTL analysis [33] showed that rs651007 could trans-regulate the expression of *TNFRSF1A* (Effect = 0.403, *P* = 1.30 × 10^-5^, LOD = 4.126); meanwhile, rs651007 was observed associated to higher serum TNF-R2 levels in a previous study [25], demonstrating that rs651007 may be involved in the regulation of TNF receptors. In addition, TNF signaling is believed playing an important role in obesity since TNFα mRNA or protein was found overexpressed in obese subjects in both experimental and epidemiological studies. Researchers also found that TNF signaling plays an important role in the insulin resistance of obesity [34,35]. Moreover, TNF-α has been demostrated to positively regulate ALP levels in various types of cells [36-39]. Taken together, all evidence suggests that the interaction between rs651007 and overweight and obesity on serum ALP levels may act through the regulation of TNF system. Further studies are needed to uncover the real mechanism of the interaction.

Our study are not only consistent with findings of previous GWA studies [15-17], but also in line with early reports that serum ALP levels varied in individuals with different ABO blood types [2,4,18], However, it is undeniable that the relatively small sample size of our GWAS stage limited us to detect SNPs with minor effects and SNPs with low MAF, which calls for further large sample size studies and consortium cooperation.

## Conclusions

In summary, our study confirmed that *ABO* locus was a major determinant for serum ALP levels in Chinese Han population. When comparing the present study with previously published findings, ethnic differences were observed across populations. More importantly, we found that overweight and obesity could modify the effects of *ABO* rs651007 polymorphism on serum ALP levels. Findings herein may furnish clinical application in prognosis control. Further studies are warranted to validate our findings and explore the potential mechanisms.

## Methods

### Study subjects

We performed a two-stage GWAS for serum ALP levels in individuals of Chinese Han origin, all of whom were recruited from Dongfeng-Tongji cohort study (DFTJ-cohort) in Hubei, China. DFTJ-cohort, which includes 27,009 retired employees from a state-owned automobile enterprise in China, was launched in 2008 and will be followed up every 5 years. Detailed information on demographics, lifestyle factors, baseline occupational and environmental exposures has been collected and a biospecimen bank (fasting blood serum, plasma, and DNA) is available as well. The main goal of the study was to identify environmental and genetic risk factors for chronic diseases, to investigate gene-environment interactions, and to find novel biomarkers for chronic diseases prediction [40].

The discovery set (GWAS stage) included 1461 cohort participants and the validation set (second stage) contained another 8830 cohort subjects. Participants who were deemed to be healthy in physical examinations were selected, and those who have self-reported severe diseases, such as stroke, coronary heart disease, cancers and diabetes mellitus, were excluded. The protocol was approved by ethics committee in Tongji Medical College and all subjects provided written informed consents.

### Alkaline phosphatase measurement

Serum ALP levels were measured by ARCHITECT Ci8200 automatic analyzer (ABBOTT Laboratories. Abbott Park, Illinois, U.S.A) using Abbott Diagnostics reagents following standard experimental procedures from the manufacturer. This measurement was accomplished by the Biochemical Laboratory of Dongfeng Central Hospital, Shiyan, Hubei, China together with the quantification of other biochemical traits [40]. The intra-assay and inter-assay coefficients of variation for ALP were 5.35% and 3.25%.

### SNP genotyping and quality control

In discovery stage, a genome-wide genotyping scan in 1461 subjects was carried out using Affymetrix Genome-Wide Human SNP Array 6.0 chips. High-quality genotyping was performed by commercial company specialized in Affymetrix SNP array genotyping following standard experimental procedures from the manufacturer. A total of 906,703 SNPs were genotyped among 1,461 subjects in which 38,446 SNPs not mapped on autosomes were excluded. After QC filtering, SNPs with MAF < 0.01 (193,732 SNPs), HWE < 0.0001 (1,332 SNPs), and SNPs call rate < 95% (17,764 SNPs) were excluded. Individuals with a call rate < 95% were also ruled out for further analysis. Finally, 1,452 subjects with 658,288 autosomal SNPs were retained for statistical analyses, with an overall call rate of 99.68%. In validation stage, SNPs were genotyped using the iPLEX system (Sequenom, Inc., San Diego, CA, USA), in which all genotyping reactions were performed in 384-well plates according to the manufacturer’s iPLEX Application Guide (Sequenom, Inc.). Each plate included four randomly selected duplicates, as well as six negative controls using double distilled water. The average concordance rate for genotyping in validation was 99.8%.

### Statistical analysis

The population structure was evaluated by PCA using the software package EIGENSTRAT 3.0 [41], and GWAS data was analyzed utilizing PLINK 1.06 [42,43]. Quantile-quantile plot was plotted by R 2.11.1 [44], Manhattan plot of -log10 *P* was generated with Haploview (v4.1) [45], and the regional association plot was generated using SNAP [46]. To infer ungenotyped SNPs [47], MACH 1.0 software [48] was applied to impute ungenotyped SNPs using LD information from the HapMap phase II database (CHB + JPT as a reference set (2007-08_rel22, released 2007-03-02)) [49]. Imputed SNPs with high genotype information content (Rsq > 0.3) were kept for the further association analysis, which was conducted using ProbABEL software [50,51] Quanto 1.2.4 was used for power calculation [52]. BMI was calculated as the individual’s body mass (kg) divided by the square of his or her height (m^2^). Individuals whose BMI was greater than or equal to 24 kg/m^2^ were defined as overweight and obese, otherwise were classed as normal weight [53-55]. Individuals who had smoked at least one cigarette per day for more than half a year no matter currently or formerly were defined as smokers, otherwise were defined as non-smokers. Those who had drunk at least once a week for more than half a year no matter currently or formerly were classed as drinkers, else were classed as non-drinkers. Prior to analyses, serum ALP level was nature log-transformed to normalize the distribution. For GWA analysis, ALP-SNP associations were tested assuming an additive genetic model using linear regression with age, gender and the top two eigenvectors in PCA being included as covariates. SNPs that met the following criteria were selected for further validation: (1) SNPs with *P* ≤ 5.0 × 10^-5^ in GWAS stage; (2) when multiple SNPs showed strong LD (r^2^ ≥ 0.8), SNP with the lowest *P* value was selected and (3) MAF ≥ 0.05. In order to extract genotype information for all reported SNPs from the present study, imputed genotype data was used for ethnic comparisons. Loci that were positive (*P* < 5.0 × 10^-8^ in GWAS stage) in either previously published studies or the present study were gathered, and representative SNPs on each locus were selected and compared. GWAMA [56] was applied to calculate heterogeneity among studies with the option of random effect correction being abled.

SNP that showed a *P* < 0.05/6 in the validation dataset or a *P* < 5 × 10^-8^ in the combined dataset is considered to be statistically significant. For gene-environment interaction analysis, we fitted linear regression models by adding an interaction term of SNPs and environmental factors: Y = α + βs*SNP + βse*SNP*environment + βe*environment + βc*C; where SNP represented SNP to be tested (in the present situation, rs651007); environmental factors included age, gender, BMI (normal/overweight and obese), smoking and drinking, which were introduced into the model separately; and C represented covariates including age and gender. We applied the false discovery rate (FDR) approach [57] to control for multiple comparisons (1 SNP × 5 environmental factors = 5 times) in the interaction analysis. Using each of the SNPs as a covariate, we performed conditional analysis on the remaining 2 SNPs on *ABO* gene for their association with ALP. The proportion of the total serum ALP variation explained by each SNP was measured by r^2^, which is the difference of the model sum of squares between models with and without the SNP of interest divided by the corrected total sum of squares of the full model. Two tagging SNPs (rs505922 and rs8176746) in *ABO* gene were used to infer ABO blood group for each individual in GWAS dataset. ABO blood group was set into a variable of integer according to the reported binding capacity between ABO antigen and ALP (A = 0, AB = 1, B = 2, O = 3) [19,30,58]. These analyses were performed using SAS version 9.2 (SAS Institute, Cary, NC).

## Competing interests

The authors declare that they have no competing interests.

## Authors’ contributions

JL participated in the genotyping, performed the statistical analysis and drafted the manuscript. MH contributed to the conception and design, involved in drafting the manuscript and revising it critically for important intellectual content. LG and YH performed the statistical analysis. CW, LZ, HG contributed to the acquisition of data and SNPs selection. JY, JZ, XM, ML, DL, HY, FBH, DL and TW have been involved in acquisition of data and contributed to conception and design. BY, XD, QD, SH, LG, DH, SD and TW have been involved in acquisition of data. All authors read and approved the final manuscript.

## Supplementary Material

Additional file 1: Table S1 Clinical characteristics of the participants in the discovery and validation datasets from DFTJ-cohort Study. Click here for file

Additional file 2: Figure S1 Q-Q plots of GWAS for ALP in discovery set. Description of data: The horizontal axis shows -log10 transformed expected P values, while the vertical axis indicates -log10 transformed observed P values. The genomic inflation factor λ is 1.011. Click here for file

Additional file 3: Table S2 Variance of serum ALP level explained by SNPs. Click here for file

Additional file 4: Table S3 Interactions between *ABO* rs651007 and drinking, smoking, gender and overweight and obesity. Click here for file
