# Supplementary material for: Genome-wide association study on serum alkaline phosphatase levels in a Chinese population
Source: BMC Genomics. 2013 Oct 5;14:684. doi: 10.1186/1471-2164-14-684 (PMC3851471; doi:10.1186/1471-2164-14-684)
Supplement: Additional file 1: Table S1 — Clinical characteristics of the participants in the discovery and validation datasets from DFTJ-cohort Study. [file 1471-2164-14-684-S1.doc]

**Additional file 1 Table S1. Clinical characteristics of the participants in the discovery and validation datasets from DFTJ-cohort Study**

|  | **GWAS Discovery Set** | **Validation Set** |
| --- | --- | --- |
| n | 1,452 | 8,830 |
| Female, n (%) | 316 (21.8) | 5,141 (58.2) |
| Age (years) | 63.05 ± 8.14 | 61.95 ± 7.83 |
| BMI (kg/m^2^) | 24.73 ± 3.33 | 24.26 ± 3.30 |
| Waist (cm) | 84.68 ± 9.57 | 82.31 ± 9.54 |
| Smoking status (n, %) |  |  |
| Non-smokers | 708(48.8) | 6,423 (73.3) |
| Smokers | 732(50.4) | 2,345 (26.7) |
| Drinking status (n, %) |  |  |
| Non-drinkers | 817 (56.3) | 6,664 (75.5) |
| Drinkers | 634 (43.7) | 2,159 (24.5) |
| Activities (hours/week)  Median (P25 - P75) ^*^ | 7 (3 - 10.5) | 7 (3 - 10.5) |
| ETS (n, %) ^#^ | 272 (19.4) | 1,508 (17.2) |
| ALP (U/L) | 90.02 ± 26.51 | 90.26 ± 35.41 |

^*^ Activities include walking, biking, taichi, jogging, swimming, dancing, climbing and ball games.

^#^ ETS is short for environmental tobacco smoke.
