# Supplementary material for: Genome-wide association study on serum alkaline phosphatase levels in a Chinese population
Source: BMC Genomics. 2013 Oct 5;14:684. doi: 10.1186/1471-2164-14-684 (PMC3851471; doi:10.1186/1471-2164-14-684)
Supplement: Additional file 2: Figure S1 — Q-Q plots of GWAS for ALP in discovery set. Description of data: The horizontal axis shows -log10 transformed expected P values, while the vertical axis indicates -log10 transformed observed P values. The genomic inflation factor λ is 1.011. [file 1471-2164-14-684-S2.doc]

**Additional file 2 Figure S1. Q-Q plots of GWAS for ALP in discovery set.** The horizontal axis shows -log10 transformed expected P values, while the vertical axis indicates -log10 transformed observed *P* values. The genomic inflation factor λ is 1.011.

**
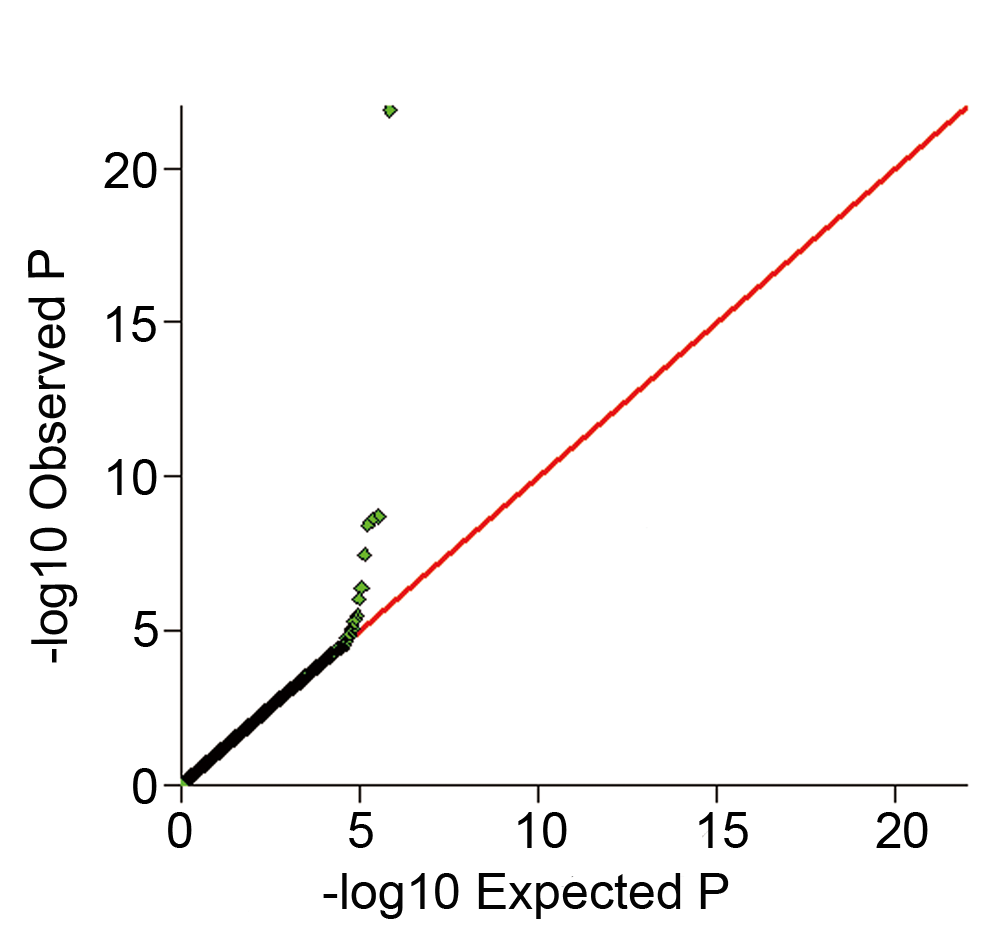
**
