# Supplementary material for: Genome-wide association study on serum alkaline phosphatase levels in a Chinese population
Source: BMC Genomics. 2013 Oct 5;14:684. doi: 10.1186/1471-2164-14-684 (PMC3851471; doi:10.1186/1471-2164-14-684)
Supplement: Additional file 3: Table S2 — Variance of serum ALP level explained by SNPs. [file 1471-2164-14-684-S3.doc]

**Additional file 3 Table S2. Variance of serum ALP level explained by SNPs**

| **Variables** | **Original values** | | **Association analysis conditioned on** **rs8176720** | | **Association analysis conditioned on rs651007** | | **Association analysis conditioned on rs7025162** | | **Association analysis including all three SNPs** | |
| --- | --- | --- | --- | --- | --- | --- | --- | --- | --- | --- |
|  | ***P* value** | **r^2^ (%) ^a^** | ***P* value** | **r^2^ (%) ^a^** | ***P* value** | **r^2^ (%) ^a^** | ***P* value** | **r^2^ (%) ^a^** | ***P* value** | **r^2^ (%) ^a^** |
| rs8176720 | 2.50×10^-21^ | 0.88 | - | - | 0.187 | 0.02 | 0.012 | 0.07 | 0.559 | 0.00 |
| rs651007 | 1.12×10^-56^ | 2.47 | 1.64×10^-30^ | 2.20 | - | - | 6.88×10^-25^ | 2.15 | 1.84×10^-23^ | 2.15 |
| rs7025162 | 2.82×10^-27^ | 1.14 | 4.92×10^-10^ | 0.99 | 0.134 | 0.03 | - | - | 0.298 | 0.03 |

**^a^** r^2^ was the difference of the model sum of squares between models with and without the interested variables divided by the corrected total sum of squares of the full model. In the present table, it represented the proportion of total serum ALP variance explained independently by each variable.
