# Supplementary material for: Genome-wide association study on serum alkaline phosphatase levels in a Chinese population
Source: BMC Genomics. 2013 Oct 5;14:684. doi: 10.1186/1471-2164-14-684 (PMC3851471; doi:10.1186/1471-2164-14-684)
Supplement: Additional file 4: Table S3 — Interactions between ABO rs651007 and drinking, smoking, gender and overweight and obesity. [file 1471-2164-14-684-S4.doc]

**Additional file 4 Table S3. Interactions between *ABO* rs651007 and drinking, smoking, gender and overweight and obesity**

|  | **ALP levels by ABO (rs651007) genotypes ^a^** | | | ***P* value ^b^** | ***P* for interaction ^c^** | **FDR for**  **interaction ^d^** |
| --- | --- | --- | --- | --- | --- | --- |
|  | **GG** | **GA** | **AA** |  |  |  |
| Drinking |  |  |  |  |  |  |
| Non-drinkers | 90.71 ± 1.36 (n=4,415) | 82.24 ± 1.35 (n=2,487) | 80.26 ± 1.33 (n=393) | 4.13×10^-38^ | 0.522 | 1 |
| Drinkers | 87.09 ± 1.33 (n=1,645) | 77.21 ± 1.35 (n=911) | 77.96 ± 1.31 (n=165) | 3.41×10^-20^ |  |  |
| Smoking |  |  |  |  |  |  |
| Non-smokers | 89.73 ± 1.36 (n=4,226) | 81.60 ± 1.36 (n=2,331) | 80.22 ± 1.34 (n=377) | 1.65×10^-34^ | 0.098 | 0.49 |
| Smokers | 89.70 ± 1.35 (n=1,789) | 79.42 ± 1.35 (n=1,031) | 78.29 ± 1.29 (n=177) | 9.19×10^-24^ |  |  |
| Gender |  |  |  |  |  |  |
| Male | 88.32 ± 1.33 (n=2,842) | 78.32 ± 1.34 (n=1,577) | 77.02 ± 1.31 (n=271) | 6.24×10^-38^ | 0.054 | 0.27 |
| Female | 91.06 ± 1.38 (n=3,217) | 83.15 ± 1.36 (n=1,822) | 82.26 ± 1.34 (n=288) | 5.33×10^-23^ |  |  |
| Overweight and obese |  |  |  |  |  |  |
| normal weight | 89.93 ± 1.36 (n=2,843) | 80.59 ± 1.37 (n=1,601) | 77.78 ± 1.32 (n=256) | 2.53×10^-33^ | 0.007 | 0.036 |
| Overweight and obese | 89.52 ± 1.35 (n=3,141) | 81.12 ± 1.34 (n=1,764) | 81.24 ± 1.34 (n=297) | 2.36×10^-25^ |  |  |

**^a^** Values are shown as mean ± SD.

**^b^** P-values for rs651007 genotypes in each stratum using linear regression model with age and gender being included as covariates.

**^c^** P-values for the interaction terms between rs651007 genotypes and drinking, smoking, gender and overweight and obesity in the interaction model.

^d^ multiple corrected *P* values of *P* for interaction by using method recommended by Benjamini and Hochberg.
